# S100A4 is elevated in axial spondyloarthritis: a potential link to disease severity

**DOI:** 10.1186/s41927-019-0110-7

**Published:** 2020-01-31

**Authors:** Barbora Šumová, Lucie Andrés Cerezo, Hana Hulejová, Klára Prajzlerová, Michal Tomčík, Kristýna Bubová, Jan Štěpán, Mária Filková, Tereza Kropáčková, Mariam Grigorian, Karel Pavelka, Jiří Vencovský, Ladislav Šenolt

**Affiliations:** 1 Institute of Rheumatology, Prague, Czech Republic; 2 Department of Rheumatology, 1st Faculty of Medicine, Charles University, Prague, Czech Republic; 3 Department of Neuroscience and Pharmacology, Faculty of Health Sciences, University of Copenhagen, Copenhagen, Denmark

**Keywords:** S100A4, Axial spondyloarthritis, Disease severity, Disease duration, Syndesmophyte

## Abstract

**Background:**

S100A4 is a member of calcium binding S100 protein family well known for its role in cancer progression and metastasis. Nevertheless, S100A4 also serves as a negative regulator of bone formation. Dickkopf-1 (DKK-1), marker of bone remodelling, is also implicated in the process of syndesmophyte formation in ankylosing spondylitis. The aim of our study was to evaluate plasma levels of S100A4 in patients with axial spondyloarthritis and to determine the potential association of S100A4 with disease severity, clinical manifestations and with bone changes in a cross-sectional study.

**Methods:**

Fifty-eight patients with axial spondyloarthritis and 40 healthy controls were studied. Biological samples were analysed for S100A4 and Dickkopf-1. Disease activity was assessed according to the Bath Ankylosing Spondylitis Disease Activity Index. C-reactive protein (CRP) was used as a marker of inflammation. Radiographic damage was assessed using the modified Stoke Ankylosing Spondylitis Spinal Score (mSASSS).

**Results:**

The plasma levels of S100A4 were significantly higher in patients with axial spondyloarthritis compared to heathy controls (*p* < 0.0001). The levels of S100A4 were higher in early stages of the disease and lower in patients with the presence of syndesmophytes (*p* = 0.009). Furthermore, we found weak but significant inverse correlation of plasma S100A4 with the mSASSS (r = − 0.363, *p* = 0.030). Levels of S100A4 were negatively associated with disease duration (r = − 0.404, *p* = 0.002) and positively with Dickkopf-1 binding capacity (r = 0.312, *p* = 0.023).

**Conclusions:**

This is the first study showing elevated circulating levels of S100A4 in patients with axial spondyloarthritis, particularly in early stages of the disease prior to spinal involvement, and its significantly lower levels in patients with syndesmophytes. The role of S100A4 in the pathogenesis of axial spondyloarthritis can be suggested.

## Background

Axial spondyloarthritis (axSpA) is a chronic inflammatory rheumatic disease predominantly affecting the axial skeleton. The early, non-radiographic phase of the disease is diagnosed by the presence of chronic back pain and active sacroiliac inflammation on magnetic resonance imaging and/or combination of other specific findings [1]. However, ankylosing spondylitis is already associated with radiographic sacroiliitis and, in some patients, with the development of syndesmophytes and extra-articular manifestations. Genetic risk associations, HLA-B27-mediated pathology and perturbations in antigen-presentation pathways may subsequently contribute to the pathogenesis of axSpA by up-regulation of various cytokines, including interleukin (IL)-23/IL-17 [2]. A hallmark of the disease is entheseal inflammation, new spinal bone formation, and bone resorption, suggesting that bone metabolism is impaired during the course of the disease [3].

S100A4 is a Ca-binding protein regulating cell growth, survival and motility, and is associated with malignancies, as well as with various fibrotic, inflammatory and autoimmune diseases [4–7]. Its role as a negative regulator of bone formation has been previously described [8]. Inhibition of S100A4 contributes to up-regulation of osteoblast-related genes in several cell lines [9, 10]. Furthermore, S100A4 deficiency was shown to be associated with higher trabecular and cortical bone mass, a reduced number of functional osteoclasts and a higher number of activated osteoblasts [11, 12]. It is well established that normal bone homeostasis is controlled by Wnt signaling [13] and that S100A4 is a target gene of the Wnt/β-catenin pathway [14]. Recent studies show that Dickkopf-1 (DKK-1), a negative regulator of Wnt/β-catenin signalling, participates in the bone remodeling and syndesmophytes formation in ankylosing spondylitis [15]. Moreover, Sack et al. demonstrated that expression of both DKK-1 and S100A4 was decreased following the Wnt/β-catenin pathway inhibition [9].

The aim of the study was to evaluate circulating levels of S100A4 in patients with axSpA and to investigate potential associations between S100A4 levels, disease severity and syndesmophyte formation in a cross-sectional study.

## Methods

### Subjects

Circulating S100A4 was analysed in a cross-sectional cohort study of 58 consecutive patients with axSpA fulfilling the Assessment of SpondyloArthritis international Society (ASAS) classification criteria for axSpA [16] that were recruited from a single centre of the outpatient department of the Institute of Rheumatology in Prague as demonstrated elsewhere [17, 18].

Twenty-one patients (11 male; age 30.6 [28.6–40.7] median [IQR]) had non-radiographic axSpA (nr-axSpA) and 17 patients (15 male, age 31.6 [27.2–37.3] median [IQR]) had ankylosing spondylitis without spinal involvement (AS I), and 20 patients (16 male, age 37.3 [32.6–40.8] median [IQR]) had ankylosing spondylitis with the presence of syndesmophytes (AS II). Fourty age- and sex-matched healthy controls (31 male, age 34.3 [30.1–38.6] median [IQR]) were enrolled in the study. Disease activity was determined using the Bath Ankylosing Spondylitis Disease Activity Index (BASDAI) that consists of a 0–10 scale measuring fatigue, spinal and joint pain, enthesitis and duration and severity of morning stiffness [19]. Radiographic damage was assessed using the modified Stoke Ankylosing Spondylitis Spinal Score (mSASSS) [20], a well-validated scoring method measuring lumbar and cervical spine chronic structural changes including erosions, sclerosis, squaring, non-bridging and bony bridging syndesmophytes (range 0–72). The occurrence and frequency of extra-articular manifestations such as uveitis, psoriasis, enthesitis, peripheral arthritis or inflammatory bowel disease were summarised in Table 1.

**Table 1:** Clinical characteristics of the patients with axial spondyloarthritis and healthy controls

		nr-axSpA (*n* = 21)	AS I (*n* = 17)	AS II (*n* = 20)	Healthy controls (*n* = 40)
Age (years)	median [IQR]	30.6 [28.6–40.7]	31.6 [27.2–37.3]	37.3 [32.6–40.8]	34.3 [30.1–38.6]
Sex (male gender)	N (%)	11 (52)	15 (88)	16 (80)	31 (72)
Disease duration^a^ (years)	median [IQR]	0.1 [0.0–5.0]	3.0 [0.7–6.0]	4.7 [3.8–9.0]	–
BASDAI (units NRS)	median [IQR]	4.8 [1.6–5.7]	7.1 [4.4–8.0]	4.5 [2.7–5.7]	–
HLA-B27 (+)	N (%)	21 (100)	14 (82)	16 (80)	–
CRP, mg/L	median [IQR]	3.2 [2.4–5.2]	6.4 [5.4–7.3]	4.5 [3.6–5.1]	–
Uveitis (+)	N (%)	9 (43)	6 (35)	8 (40)	–
Psoriasis (+)	N (%)	0 (0)	0 (0)	0 (0)	–
Enthesitis (+)	N (%)	13 (62)	1 (6)	7 (35)	–
IBD (+)	N (%)	1 (5)	0 (0)	0 (0)	–
Peripheral arthritis (+)	N (%)	18 (86)	10 (59)	15 (75)	–
NSAIDs	N (%)	15 (71)	13 (77)	9 (45)	–
csDMARDs	N (%)	6 (29)	3 (18)	0 (0)	–
TNF inhibitors	N (%)	0 (0)	1 (6)	11 (55)	–

*nr-axSpA* Non-radiographic axial spondyloarthritis, *AS I* Ankylosing spondylitis without spinal involvement, *AS II* Ankylosing spondylitis with the presence of syndesmophytes, *BASDAI* The Bath Ankylosing Spondylitis Disease Activity Index, *NRS* Numeric Rating Scale, *csDMARDs* Conventional synthetic disease-modifying antirheumatic drugs, *CRP* C-reactive protein, *IBD* Inflammatory bowel disease, *IQR* Interquartile range, *N* Number of individuals, *NSAIDs* Non-steroidal anti-inflammatory drugs, *TNF* Tumor necrosis factor

^a^since diagnosis

The patients were recruited from the outpatient department of rheumatology and healthy controls were recruited from the employees of the Institute of Rheumatology in Prague. Written informed consent was obtained from all participants prior to enrolment and the study was approved by the local ethics committee at the Institute of Rheumatology. Demographic and clinical characteristics of the patients are summarised in Table 1.

### Laboratory measurements

Circulating levels of S100A4 were measured using a homemade ELISA as previously described [7], and Dickkopf-1 (DKK-1) levels were measured by commercial ELISA (Biomedica, Vienna, Austria) according to the manufacturer’s protocol. DKK-1 binding capacity to its receptor (LRP6) was measured. Briefly, the ELISA plates were coated with 3 μg/mL of recombinant human LRP-6/Fc chimaera (R&D Systems, Minneapolis, MN, Canada) prior to the addition of samples and detection was performed using human anti-DKK-1 antibody (R&D Systems, Minneapolis, MN, Canada). An immuno-turbidimetric technique was used to measure CRP levels using an Olympus Biochemical Analyzer (Olympus CO Ltd.,Tokyo, Japan).

### Statistical analysis

Differences in S100A4 levels between the groups were analysed using the Mann-Whitney *U*-test and analysis of covariance. The Spearman test was used for correlation between S100A4 and clinical and laboratory parameters. The analysis was adjusted for confounders including disease duration, sex, age, BASDAI and CRP using the partial correlation method. Data were analysed using STATISTICA software (Version 12, 2013 Edition; Statsoft Inc., Tulsa, OK, USA). *P*-values less than 0.05 were considered statistically significant. The data were expressed as the median (interquartile range, IQR).

## Results

### Higher plasma levels of S100A4 in patients with axSpA

**Fig. 1:**
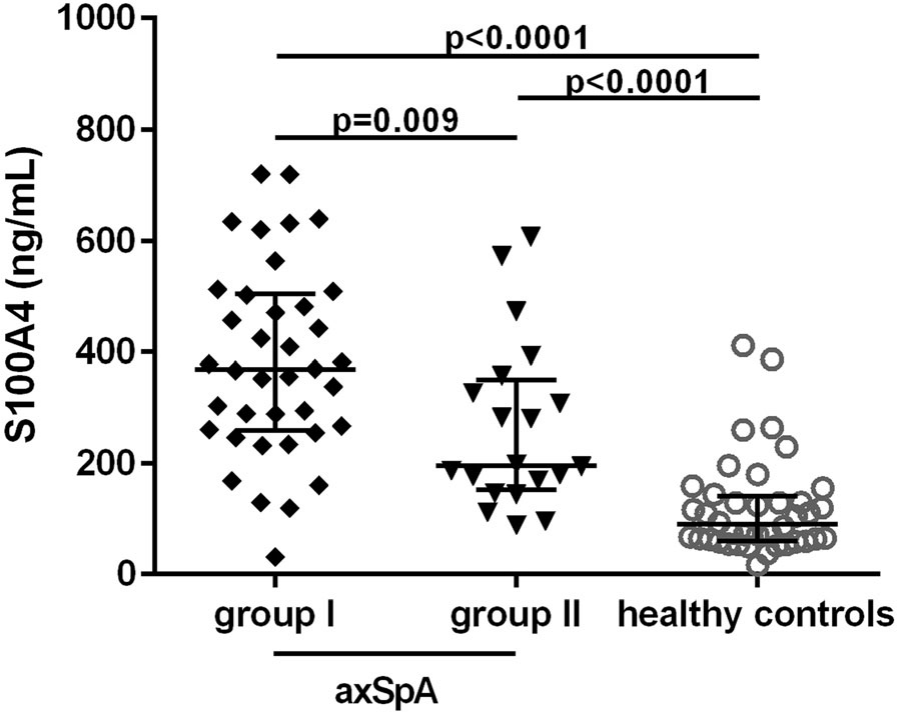
Increased circulating levels of S100A4 in axSpA patients. The levels of plasma S100A4 are higher in patients with axial spondyloarthritis (axSpA) compared to healthy controls and in axSpA patients without syndesmophytes (nr-axSpA + AS I) compared to those with the presence of syndesmophytes (AS II). Horizontal bars show the median with whiskers representing the interquartile range (IQR). AS I, ankylosing spondylitis without spinal involvement; AS II, ankylosing spondylitis with the presence of syndesmophytes

The plasma levels of S100A4 were significantly higher in patients with axSpA compared to healthy controls (median [IQR]: 317.0 [192.2–471.0] vs. 89.7 [60.5–140.1] ng/mL; *p* < 0.0001). The levels of S100A4 were significantly lower in axSpA patients with more bone formation, as demonstrated by the presence of syndesmophytes, compared to axSpA patients with no spinal involvement (median [IQR]: 196.1 [151.7–349.9] vs. 368.3 [259.4–504.1] ng/mL; *p* = 0.009, Fig. 1). However, when adjusted for disease duration, sex, age, BASDAI and CRP levels, the *p*-value reached the border of the statistical significance (*p* = 0.062). Furthermore, there was no difference in the levels of plasma S100A4 between patients with nr-axSpA and ankylosing spondylitis without syndesmophytes (369.8 [240.1–536.4] vs. 366.8 [275.1–449.8] ng/mL; *p* = 0.921).

### Plasma levels of S100A4 are associated with radiographic damage and disease duration

**Fig. 2:**
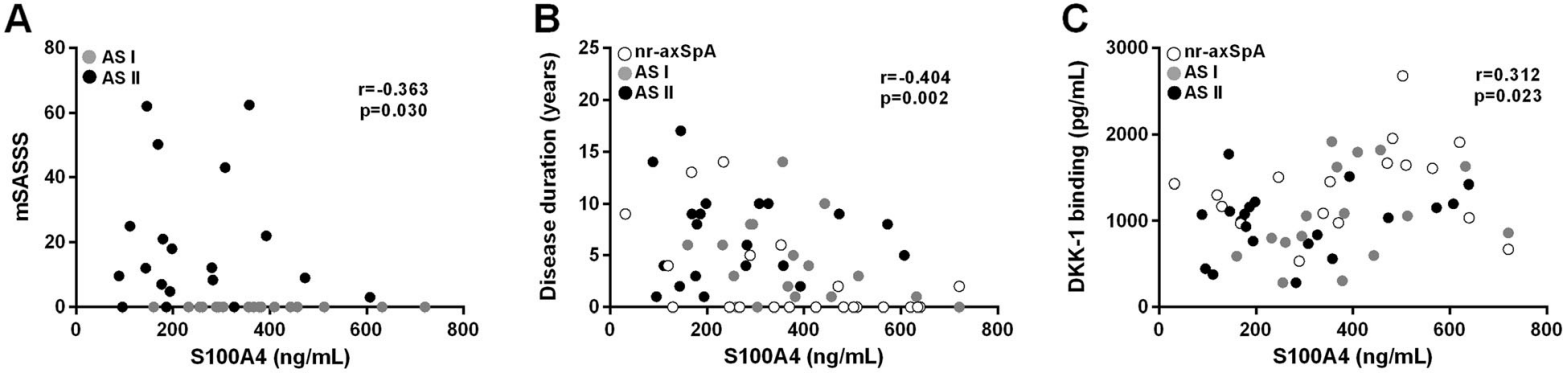
Association of S100A4 levels with clinical and laboratory parameters. S100A4 levels are inversely correlated with the modified Stoke Ankylosing Spondylitis Spine Score (mSASSS) (**a**) and disease duration (**b**) and are positively correlated with DKK-1 binding capacity (**c**)

We found a weak inverse correlation of S100A4 levels in plasma with the mSASSS (r = − 0.363, *p* = 0.030; Fig. 2a). Although there was no association between S100A4 and age or gender, the S100A4 levels negatively correlated with disease duration (r = − 0.404, *p* = 0.002; Fig. 2b). In contrast, S100A4 levels did not correlate with disease activity as determined by BASDAI or CRP levels (r = − 0.197, *p* = 0.139; r = − 0.219, *p* = 0.099; respectively) and did not differ between patients with or without peripheral joint involvement or other clinical manifestations such as psoriasis, inflammatory bowel disease or presence of uveitis (Additional file 1). Furthermore, S100A4 plasma levels did not differ between patients treated with NSAIDs, csDMARDs or TNF inhibitors (Additional file 2).

### Plasma levels of S100A4 correlate with DKK-1 binding capacity in patients with axSpA

Since S100A4 levels were inversely associated with spinal bone formation, we were curious to determine the association between S100A4 and DKK-1. Interestingly, the DKK-1 binding capacity, but not DKK-1 levels themselves, exhibited weak but significant association with the levels of S100A4 (r = 0.312, *p* = 0.023, Fig. 2a).

## Discussion

The present study found elevated plasma levels of S100A4 in patients with, particularly in those in early stages and without the presence of syndesmophytes. Moreover, S100A4 levels were inversely correlated with radiographic spinal damage. Finally, S100A4 levels positively correlated with DKK-1 binding capacity, suggesting its involvement in bone changes during the pathogenesis of axSpA.

The role of S100A4 has already been demonstrated in several autoimmune disorders [5–7, 21–26]. We and others found local or systemic up-regulation of S100A4 in rheumatoid arthritis (RA), psoriatic arthritis, idiopathic inflammatory myopathies or in systemic sclerosis and showed its association with disease severity [5, 6, 21, 23, 24]. Similar to systemic lupus erythematosus [26], levels of S100A4 in plasma of axSpA patients are markedly lower compared to patients with RA [7]. It could be due to the local overproduction of S100A4 in the RA joints and its subsequent up-regulation in the circulation. In addition to various immune cells, proliferating fibroblasts were described as important producers of S100A4 [5, 7, 25]. Fibroblasts are the most prevalent connective tissue cells in ligaments and entheses and are shown to be closely associated with ligament ossification [27]. We therefore assume that the elevated S100A4 levels in patients with axSpA are produced by both immune cells and activated fibroblasts at sites of entheseal inflammation, particularly at early stages of the disease. Indeed, patients at early stages of the disease exhibited higher levels of circulating S100A4 compared to patients with longer disease duration and presence of syndesmophytes. This was in line with our finding of the inverse association of S100A4 to the disease duration and to the spinal bone formation occurring over time. Several authors reported an up-regulated expression or synthesis of S100A4 in osteoblast progenitors and its consequent decline in mature osteoblasts and in osteocytes [8, 28]. These studies could at least partly explain the decrease of S100A4 during the disease progression towards the formation of syndesmophytes. On the other hand, the overall elevation of S100A4 in axSpA patients is in contrast to the reports describing the increased bone formation [12] or reduced number of functional osteoclasts [11] in S100A4 knockout mice. Nevertheless, it is of note that the abovementioned studies [11, 12] did not work with the mouse model of ankylosing spondylitis. Therefore, one can only speculate about the possible effect of S100A4 depletion on the bone formation in the conditions of experimentally induced autoimmune disease.

In this study, we also found a positive correlation between DKK-1 binding ability and S100A4 levels. DKK-1 is known as a natural regulatory molecule of Wnt signalling, which is downregulated in ankylosing spondylitis and was shown to be crucial for the development of new syndesmophytes [15]. DKK-1 inhibition prevented the formation of bone erosions in a mouse model of TNF transgenic mouse model of inflammatory arthritis, although bone formation in peripheral joints was not affected [29]. Furthermore, combined inhibition of DKK-1 and TNF led to a complete abrogation of osteoclast formation and to increased numbers of osteoblasts, bone formation and ankylosis of the sacroiliac joint [30]. The positive association of S100A4 with the DKK-1 binding capacity and the obvious functional parallel of S100A4 and DKK-1 could indicate that they may be co-regulated. This is further supported by the fact that both S100A4 and DKK-1 are prominent target genes of Wnt/β-catenin signalling pathway and their expression is down-regulated following the Wnt/β-catenin pathway inhibition [9]. Our results uncover the potential value of S100A4 and DKK-1 for assessing the syndesmophyte formation in axSpA. However, larger and prospective studies are needed to further investigate the exact functional role of S100A4 in the molecular interplay underlying the bone metabolism and in the new bone formation in patients with axSpA. In conclusion, S100A4 levels are elevated in patients with axSpA, particularly in those at early stages without the presence of syndesmophytes and may be involved in the altered bone formation.

## Conclusion

Elevated S100A4 levels can be found in patients with axSpA, particularly in those at early stages and without the presence of syndesmophytes. In addition, excessive spinal bone formation occurring over time seems to be reflected by the decline in S100A4 levels. Thus, S100A4 may be implicated in the pathological process of the altered bone formation in axSpA.

## Supplementary information

**Additional file 1.** Circulating levels of S100A4 in axSpA patients based on clinical characteristics; IBD, Inflammatory bowel disease; N, number of individuals. Data are presented as median and interquartile range.

**Additional file 2.** Levels of S100A4 in plasma of axSpA patients depending on the therapy. The levels of circulating S100A4 are comparable among the axSpA patients on different therapies. NSAIDs, non-steroidal anti-inflammatory drugs; TNF, tumor necrosis factor; csDMARDs, conventional synthetic disease-modifying antirheumatic drugs. Horizontal line represents median.

## Data Availability

The datasets used and/or analysed during the current study are available from the corresponding author on reasonable request**.**

## Abbreviations

ASAS: The Assessment of SpondyloArthritis international Society
axSpA I: Axial spondyloarthritis without syndesmophytes
axSpA II: Axial spondyloarthritis with the presence of syndesmophytes
axSpA: Axial spondyloarthritis
BASDAI: The Bath Ankylosing Spondylitis Disease Activity Index
CRP: C-reactive protein
csDMARDs: Conventional synthetic disease-modifying antirheumatic drugs
DKK-1: Dickkopf-1
IBD: Inflammatory bowel disease
IQR: Interquartile range
mSASSS: The modified Stoke Ankylosing Spondylitis Spinal Score
N: Number of individuals
nr-axSpA: Non-radiographic axial spondyloarthritis
NRS: Numeric Rating Scale
NSAIDs: Nonsteroidal anti-inflammatory drugs
NSAIDs: Non-steroidal anti-inflammatory drugs
TNF: Tumour necrosis factor
