# Supplementary material for: S100A4 is elevated in axial spondyloarthritis: a potential link to disease severity
Source: BMC Rheumatol. 2020 Jan 31;4:13. doi: 10.1186/s41927-019-0110-7 (PMC6993388; doi:10.1186/s41927-019-0110-7)
Supplement: Supplementary file 1 — Additional file 1. Circulating levels of S100A4 in axSpA patients based on clinical characteristics; IBD, Inflammatory bowel disease; N, number of individuals. Data are presented as median and interquartile range. [file 41927_2019_110_MOESM1_ESM.docx]

**Additional file 1.** **Circulating levels of S100A4 in axSpA patients based on clinical characteristics;** IBD, Inflammatory bowel disease; N, number of individuals. Data are presented as median and interquartile range.

|  | |  | | |  | |  | |  | |
| --- | --- | --- | --- | --- | --- | --- | --- | --- | --- | --- |
|  | | | **N (%)** | | **POSITIVE** | | **NEGATIVE** | | **p-value** | |
| **Uveitis** | | 23 (40) | | | 267.3 [160.4 - 472.6] | | 368.3 [243.3 - 478.5] | | 0.109 | |
| **Psoriasis** | | 0 | | | - | | - | | - | |
| **IBD** | | 1 (2) | | | 31.6 [31.6 - 31.6] | | 196.1 [338.3 - 477.1] | | NA | |
| **Peripheral arthritis** | | 43 (74) | | | 307.6 [179.2 - 481.7] | | 372.5 [273.5 - 460.2] | | 0.507 | |
| **Enthesitis** | | 21 (36) | | | 332.4 [192.2 - 517.8] | | 330.2 [190.6 - 467.0] | | 0.672 | |
|  |  | | |  | |  | |  | |  |
