# Supplementary material for: S100A4 is elevated in axial spondyloarthritis: a potential link to disease severity
Source: BMC Rheumatol. 2020 Jan 31;4:13. doi: 10.1186/s41927-019-0110-7 (PMC6993388; doi:10.1186/s41927-019-0110-7)

**Additional file 2. Levels of S100A4 in plasma of axSpA patients depending on the therapy**

The levels of circulating S100A4 are comparable among the axSpA patients on different therapies. NSAIDs, non-steroidal anti-inflammatory drugs; TNF, tumor necrosis factor; csDMARDs, conventional synthetic disease-modifying antirheumatic drugs. Horizontal line represents median.


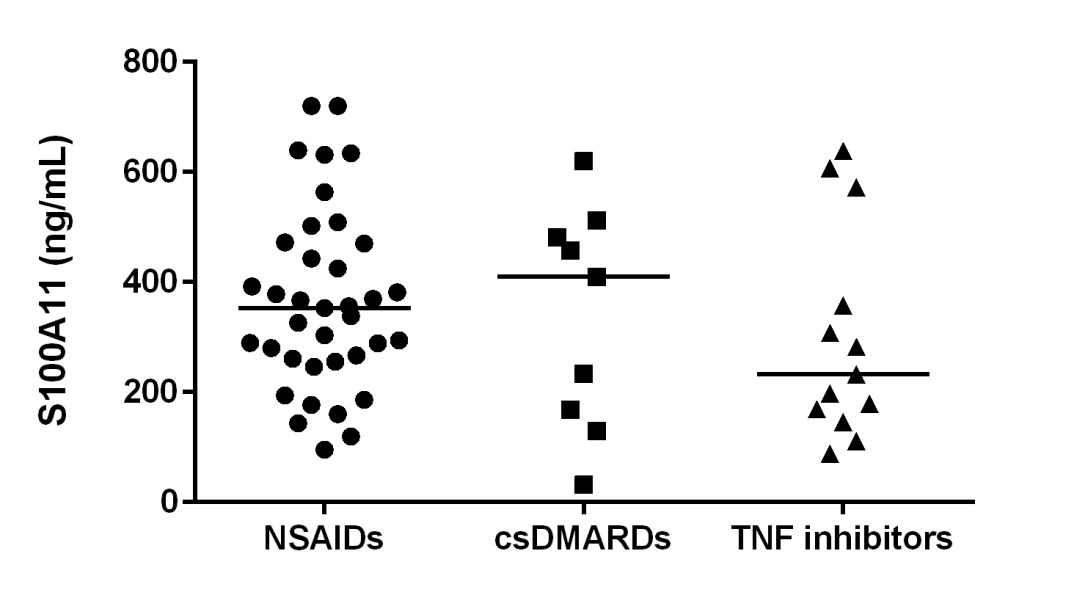

Supplement: Supplementary file 2 — Additional file 2. Levels of S100A4 in plasma of axSpA patients depending on the therapy. The levels of circulating S100A4 are comparable among the axSpA patients on different therapies. NSAIDs, non-steroidal anti-inflammatory drugs; TNF, tumor necrosis factor; csDMARDs, conventional synthetic disease-modifying antirheumatic drugs. Horizontal line represents median. [file 41927_2019_110_MOESM2_ESM.docx]
